# Tissue characterization of benign cardiac tumors by cardiac magnetic resonance imaging, a review of core imaging protocol and benign cardiac tumors

**DOI:** 10.3389/fcvm.2023.1009411

**Published:** 2023-06-27

**Authors:** Imran Haider, Hameed Ullah, Mishaim Fatima, Muhammad Sikandar Karim, Furqan Ul Haq, Abdul Majid, Muhammad Saad Anwar, Fatima Kausar Nawaz, Ijaz Ali, Atif Hussain Sarwar, Muhammad Tayyab Anwar, Abdul Wali Khan, Omama Humayun, Fazal Alam

**Affiliations:** ^1^ Radiology Department, Saint Luke’s Hospital, Chesterfield, MO, United States; ^2^ Internal Medicine Department, Hayatabad Medical Complex (HMC), Peshawar, Khyber Pakhtunkhwa, Pakistan; ^3^ Shifa Medical College, Islamabad, Pakistan; ^4^ Internal Medicine Department, King Edward Medical University, Lahore, Punjab, Pakistan; ^5^ Internal Medicine Department, Mayo Hospital, Lahore, Punjab, Pakistan; ^6^ Department of Radiation Oncology, Shifa International Hospital, Islamabad, Pakistan; ^7^ Sheikh Zayed Medical College, Pakistan; ^8^ Khyber Girls Medical College, Peshawar, Pakistan; ^9^ Internal Medicine Department, Shaikh Khalifa Bin Zayed Al-Nahyan Medical and Dental College Hospital: Shaikh Zayed Hospital, Lahore, Pakistan; ^10^ Internal Medicine Department, Khyber Teaching Hospital, Peshawar, Khyber Pakhtunkhwa, Pakistan; ^11^ Internal Medicine Department, Gujranwala Medical College, Gujranwala, Punjab, Pakistan; ^12^ Internal Medicine Department, Cook County Health and Hospitals System, Chicago, IL, United States; ^13^ Internal Medicine Department, School of Medicine, University of Missouri–Kansas City, Kansas City, United States

**Keywords:** benign cardiac tumors, magnetic resonance imaging, tissue characterization, atrial myxoma, fibroelastoma, cardiac MRI (CMR)

## Abstract

Generally, cardiac masses are initially suspected on routine echocardiography. Cardiac magnetic resonance (CMR) imaging is further performed to differentiate tumors from pseudo-tumors and to characterize the cardiac masses based on their appearance on T1/T2-weighted images, detection of perfusion and demonstration of gadolinium-based contrast agent uptake on early and late gadolinium enhancement images. Further evaluation of cardiac masses by CMR is critical because unnecessary surgery can be avoided by better tissue characterization. Different cardiac tissues have different T1 and T2 relaxation times, principally owing to different internal biochemical environments surrounding the protons. In CMR, the signal intensity from a particular tissue depends on its T1 and T2 relaxation times and its proton density. CMR uses this principle to differentiate between various tissue types by weighting images based on their T1 or T2 relaxation times. Generally, tumor cells are larger, edematous, and have associated inflammatory reactions. Higher free water content of the neoplastic cells and other changes in tissue composition lead to prolonged T1/T2 relaxation times and thus an inherent contrast between tumors and normal tissue exists. Overall, these biochemical changes create an environment where different cardiac masses produce different signal intensity on their T1- weighted and T2- weighted images that help to discriminate between them. In this review article, we have provided a detailed description of the core CMR imaging protocol for evaluation of cardiac masses. We have also discussed the basic features of benign cardiac tumors as well as the role of CMR in evaluation and further tissue characterization of these tumors.

## 1. Introduction

Cardiac magnetic resonance (CMR) imaging has become a highly valuable non-invasive technique in the diagnosis of primary and secondary cardiac tumors. CMR imaging offers an incremental value to the confirmation of the presence of a cardiac mass due to larger field of view, better contrast resolution and its unique ability to differentiate the lesion based on the tissue characterization. It also helps to confirm the localization, extent of involvement as well as functional consequences of a particular lesion. The recent development of increasing magnet strengths, surface coil channels, rapid k-space sampling, post processing methods and tissue characterization techniques have enabled CMR to emerge as an extremely helpful imaging modality in the evaluation of complicated cardiac conditions like cardiac masses. The information provided by cardiac MRI is extremely important not only for the definitive diagnosis of cardiac masses but also for determination of prognosis and planning for the management strategy (1). We are presenting a 2-part review of the utility of cardiac magnetic resonance imaging (MRI) for tissue characterization of cardiac masses. In Part 1 of the review, we will provide a detailed description of the core MRI protocol for evaluation of cardiac masses as well as illustrate the different imaging characteristics of primary benign tumors on CMR. In Part 2, we will focus on CMR characteristics of pseudo-tumors, malignant cardiac tumors, and metastatic lesions (Table 1).

**Table 1:** CMR features of primary benign, malignant tumors and secondary metastatic tumors.

Tumor	Morphology	Most common locations	Cine sequence	T1-weighted	T2-weighted	T1Weighted fat saturation pulse	First pass perfusion	Late gadolinium enhancement	Signal composition
Primary benign tumors									
Myxoma	Smooth, oval, lobular, pedunculated	Left atrium, interatrial septum near fossa ovalis	Mobile, hyperintense	Isointense/ Hypointense	Hyperintense	No	None/Mild patchy enhancement	Moderate to high	Heterogeneous
Lipoma	Smooth, broad based	Any Chamber	May be mobile	Hyperintense	Isointense/ Hyperintense	Signal drops	None	None	Homogenous
Papillary fibroelastoma	Smooth, pedicle	Valvular	Mobile, hypointense	Isointense	Hyperintense	No	None	Variable (none to high)	Homogenous
Fibroma	Smooth, encapsulated, central calcification	Ventricle near interventricular septum, intramyocardial	Immobile, variable	Isointense/ Slight hyperintense	Hypointense	No	None	Intense enhancement	Homogenous
Rhabdomyoma	Smooth, spiderlike appearance, multiple	Intramyocardial usually mobile, hypointense		Isointense/ Hypointense	Hyperintense	No	None	No/Minimal	Homogenous
Hemangioma	Lobular	Any chamber	Usually mobile	Isointense/ Hyperintense	Hyperintense	No	Yes	High (except cavernous hemangioma)	Heterogeneous
Primary malignant tumors									
Angiosarcoma	Lobular, broad based, invasive	Right atrium	Intramyocardial	Isointense/ Heterogenous	Hyperintense, heterogenous	No	Yes	High	Heterogeneous
Undiffentiated sarcoma	Lobular, broad based	Left atrium	Intramyocardial	Isointense/ Hypointense	Isointense	No	(+)?	Yes	Heterogeneous
Rhabdomyosarcoma	Lobular, invasive	Left atrium	Intramyocardial	Isointense	Hyperintense	No	(+)?	Yes	Homogeneous
Other sarcomas	Lobular, broad based	Left atrium	Intramyocardial	Isointense/ Hypointense	Hyperintense	No	(+)?	Yes	Heterogeneous (variable)
Primary cardiac lymphoma	Lobular, multiple	Right atrium	Intramyocardial	Isointense	Isointense	No	(+)?	No/Minimal	Homogenous
Secondary metastatic tumors	Lobular, smooth, invasive, multiple	Any chamber	Intramyocardial	Hypointense (exception of melanoma)	Hyperintense	No	Yes	High	Heterogeneous

Primary cardiac tumors are uncommon conditions with an estimated prevalence of 0.002%–0.3% per autopsy data and 0.15% in echocardiographic series (2). Although cardiac tumors are rare entities, their clinical significance cannot be neglected because even benign cardiac tumors can have critical hemodynamic consequences and can provide a substrate for thromboembolic events and arrhythmias (3).

Benign cardiac tumors constitute almost 70%–75% of primary cardiac tumors (Table 2). Top three tumors in adults are myxomas (30%–50%), papillary fibroelastomas (15%–20%) and lipomas (10%–15%) (4). In children, rhabdomyomas are the most common tumor type. Malignant tumors constitute approximately 25%–30% of all primary cardiac tumors. In adults the most common are angiosarcomas (10%), whereas in children the most common are rhabdomyosarcomas (5%–6%) (5). Metastatic cardiac tumors are 20–40 times more common than primary cardiac tumors and most commonly involve the pericardium (6).

**Table 2:** Approximate incidence of primary benign, malignant tumors and secondary metastatic tumors.

Tumor	Incidence
**Benign Tumors**	**70%–75%**
Myxoma	30%–50%
Lipoma	15%–20%
Fibroelastoma	10%–15%
Fibroma	5%
Hemangioma	3%–5%
Rare benign tumors	5%
**Malignant Tumors**	**25%–30%**
Angiosarcoma	10%
Rhabdomyosarcoma	5%
Mesothelioma (pericardial)	4%
Fibrosarcoma	3%
Other sarcomas	3%
Cardiac lymphoma	2%
Others	<1%
**Metastatic Tumors**	**20–40 times more common**
Lung, breast, lymphoma, melanoma	

Pseudo-tumors or “tumor-like” conditions are considered to be the most common type of cardiac masses and include intracardiac thrombus, pericardial cysts or misinterpreted normal anatomic variants (7). Therefore, detailed evaluation of a suspected cardiac mass by CMR imaging is critical for establishing the correct diagnosis as well as guiding the staging, prognosis, and management strategy. Based on the information provided by the CMR imaging unnecessary surgery can be avoided if otherwise not indicated, owing to better tissue characterization of a suspected cardiac mass.

Clinical presentations of cardiac tumors are highly variable, largely depending on the tumor size, location, and their functional consequences. Some of the tumors remain clinically silent and diagnosed incidentally on routine echocardiograms, whereas others can present with symptoms related to intra-cardiac obstruction, systemic embolization and/or arrhythmias. Generally, asymptomatic, small benign tumors do not require surgical intervention and can be managed with careful observation only. Large, symptomatic benign lesions are usually treated with complete surgical resection and most of these patients tolerate the surgery very well. Overall, patients with benign tumors have almost the same survival as that of the general population. On the other hand, malignant tumors usually have a grave prognosis; however, latest developments in the field of CMR imaging techniques have led to early detection and improved management of malignant tumors (8).

## 2. Imaging modalities for cardiac mass evaluation

Transthoracic echocardiography (TTE) is usually the first and most common diagnostic test performed in evaluation of a suspected cardiac mass (Table 3). TTE illustrates characteristic anatomical and functional features, which readily permit a differential diagnosis of a cardiac mass. Although widespread availability of echocardiography is a major advantage, there are also several well-described limitations of this technique including: operator dependence, poor tissue characterization and acquisition window restriction (especially in patients with chronic lung conditions or with a large body habitus).

**Table 3:** Imaging modalities for cardiac mass evaluation.

2-D TTE	3-D TTE, TEE, Contrast echocardiogram	CT	CMR
Most common may overcome some of useful gold standard the limitations			
Operator dependent better estimation of size, alternative to excellent contrast shape, mobility CMRI resolution			
Poor tissue radiation superior tissue characterization exposure characterization			
Acoustic window	Combined evaluation restrictions (Obese, chronic of morphology, lung disease) composition, perfusion and LGE		

2-DTTE, 2-dimensional transthoracic echocardiogram; 3-D TTE, 3-dimensional transthoracic echocardiogram; CT, computed tomography; CMR, cardiac magnetic resonance; LGE, late gadolinium enhancement; TEE, transesophageal echocardiogram.

Other imaging modalities like trans-esophageal echocardiography (TEE), 3-dimensional echocardiography and contrast echocardiography may improve some of these limitations. They provide better estimation of size, shape, mobility and functional impact of the cardiac mass. Although TEE can offer additional imaging planes, its role for the characterization of the cardiac masses is still limited because of the invasive nature of the study.

Several advancements in the computed tomography (CT) including sub-millimeter detector arrays, increased rows of detectors, half-scan post-processing algorithms, and electrocardiographic (EKG) gating have enabled this technique as an alternative to MRI in the diagnosis of cardiac masses (9). Additionally, CT can also provide information regarding vascularity of the mass (with demonstration of contrast enhancement), presence of fat and presence of calcification. Limitations of CT include the exposure to ionizing radiation (8–14 m Sv), lower temporal resolution compared with echocardiography or CMR and lower soft-tissue contrast resolution compared with CMR (10).

Positron emission tomography (PET) can also be helpful to characterize cardiac masses, but its availability still remains limited. Moreover, PET also has limited spatial resolution and it can only be a valuable tool in combination with the anatomic information provided by CT. With this combination, PET has established its role in various oncologic conditions as well as discrimination of benign from malignant lesions (11).

**Figure 1:**
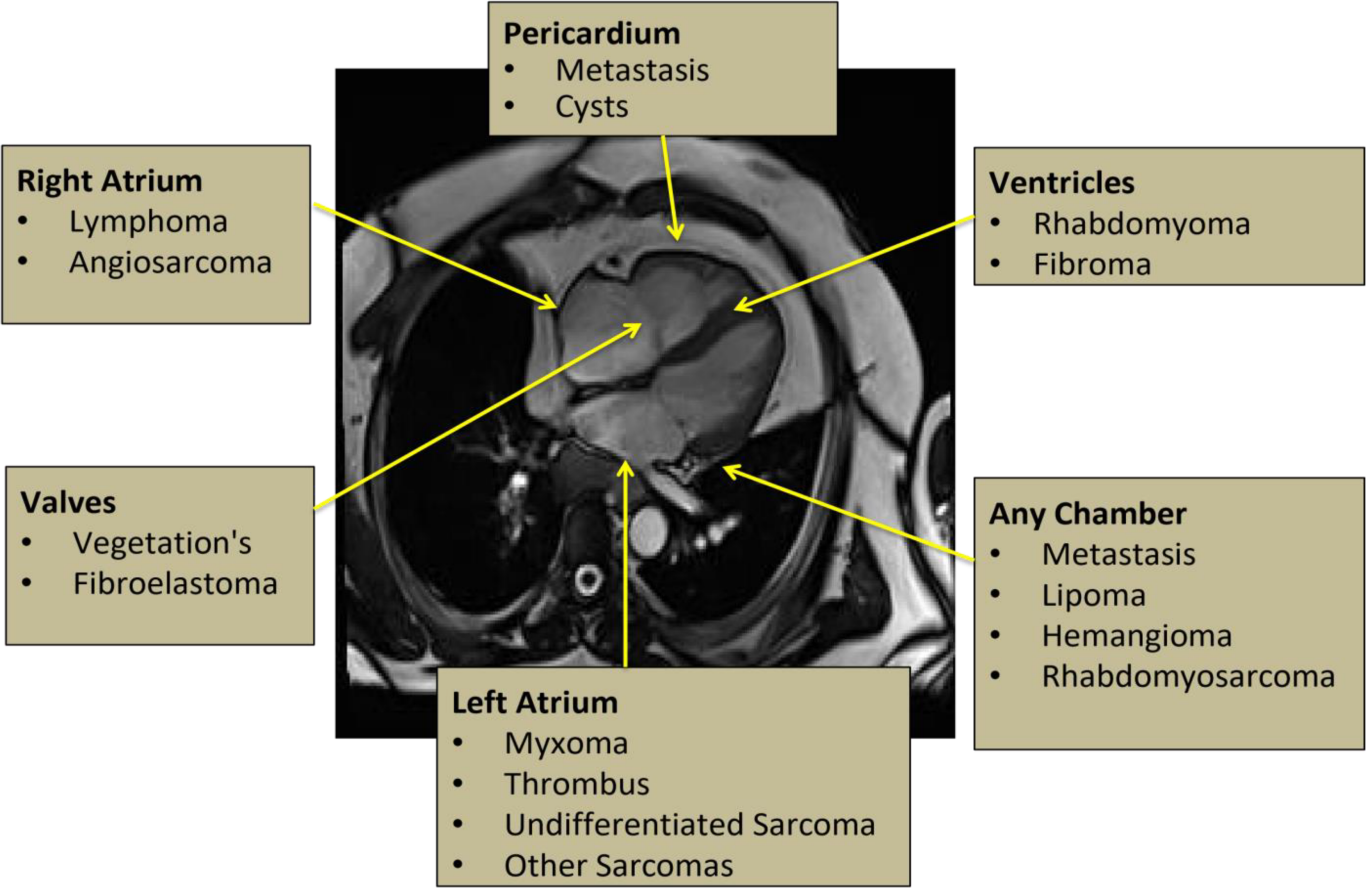
Most common locations of cardiac tumors.

Based on several consensus statements, CMR is now considered to be the “Gold Standard” imaging modality for evaluation of cardiac tumors (12). CMR has excellent contrast resolution and superior tissue characterization as compared with other modalities described above. Additionally, there is no risk of radiation exposure with CMR. The combined evaluation of location (Figure 1), morphology, composition, perfusion, and contrast uptake on late gadolinium enhancement (LGE) images makes it a diagnostic test of choice in cardiac mass evaluation. However, CMR still remains less available than other imaging modalities in most of the institutions.

Traditionally, CMR is considered contraindicated in patients with pacemakers and intracardiac defibrillators; however, most of these patients can still undergo MRI with the adequate precautions and proper expertise (13). Moreover, numbers of MR-compatible implantable devices are available in the market now (14).

Other limitations of the CMR include the need for EKG and respiratory gating. EKG gating may be a problem in patients with arrhythmias and may cause significant artifacts during image acquisition (15). Respiratory gating may be an issue in sicker patients who are not able to hold their breath, as most of the CMR sequences require breath holds to achieve better image quality. With the advancements in the field of CMR, respiratory navigator tracking methods can now be used to acquire good-quality images during free breathing.

### 2.1. A core imaging protocol for cardiac mass evaluation

Intracardiac masses vary significantly in their presentation; therefore, any standardized protocol needs to be modified according to the specific lesion. A team approach including a radiologist, cardiologist and experienced technicians is ideally required to obtain optimal imaging planes and sequences and to fully characterize the lesion. Generally cardiac tumor imaging protocols can be completed in approximately 45–60 min. Direct supervision through a team approach has the potential advantage of reducing imaging times because decisions about omitting some parts of the protocol according to the nature of a particular mass can be made at the scanner.

During cine imaging using steady state free precession (SSFP) pulse sequences, a cardiac mass should be identified in at least two orthogonal planes. For better visualization, it should be further characterized with various pulse sequence acquisitions by using the imaging plane that best visualizes the lesion.

**Figure 2:**
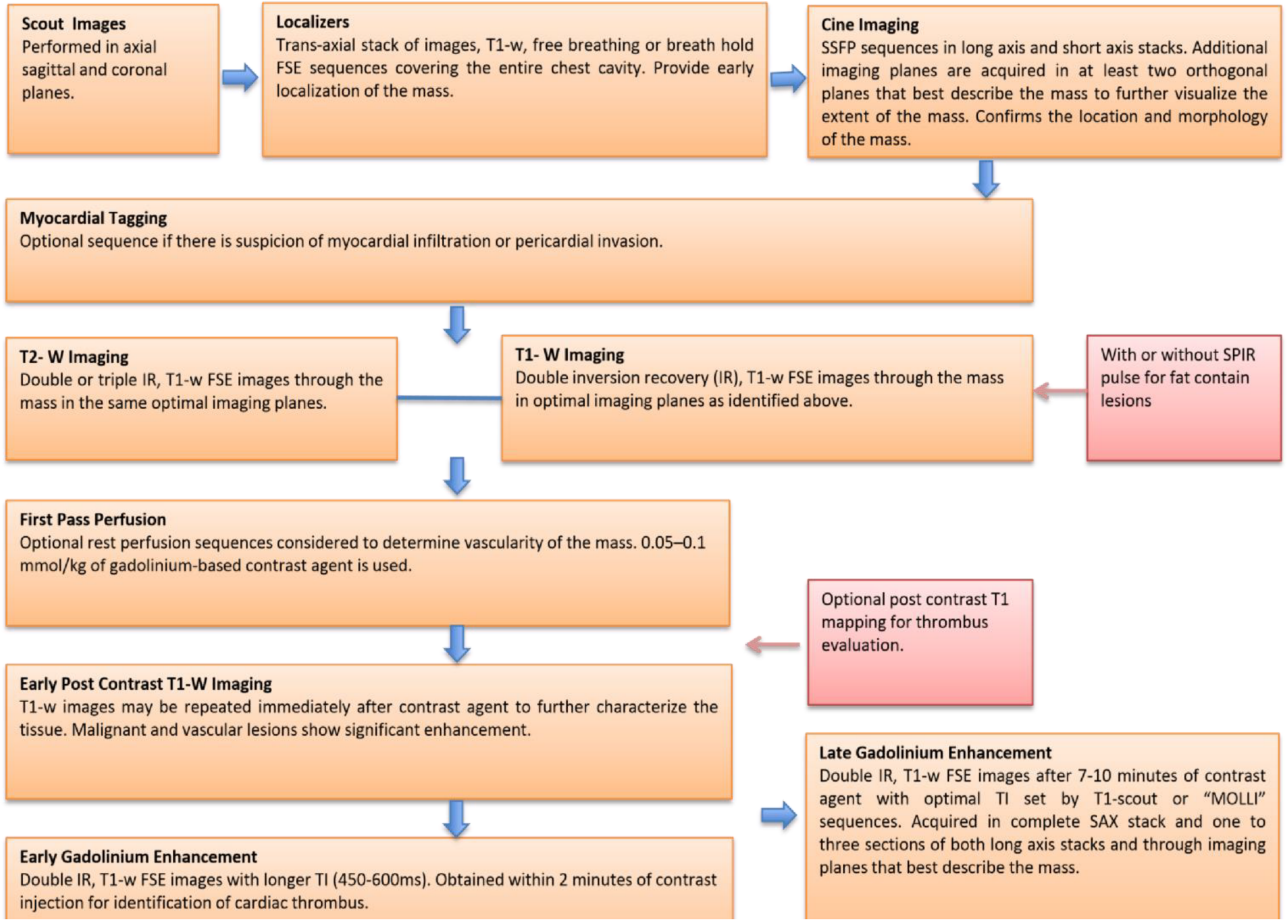
A core imaging protocol for cardiac mass evaluation. FSE, ast spin echo; IR, inversion recovery; MOLLI, modified look locker inversion recovery; SSFP, steady state free procession; SPIR, saturation spectral pre-saturation inversion recovery; TI, inversion time.

A standard imaging protocol for the evaluation of cardiac masses includes: dark blood double inversion recovery (IR) T1-weighted imaging sequences before and after contrast enhancement, dark blood double IR T1-weighted imaging with and without fat saturation, double IR T2-weighted imaging, myocardial tissue tagging (optional if suspecting myocardial infiltration or pericardial invasion), first-pass perfusion imaging (to determine the vascularity of the mass), early gadolinium enhancement with long inversion time (for thrombus evaluation) and LGE imaging. Below is the description of our standard imaging protocol for evaluation of cardiac masses (Figure 2).

#### 2.1.1. Scout images

Scout images are performed in the axial, sagittal and coronal planes. The sagittal scout images are particularly helpful to confirm the correct position of the heart at the center of the surface coil.

#### 2.1.2. Localizers

A T1-weighted trans axial stack of images covering the entire thorax is performed to localize the cardiac mass in the chest cavity This is an extremely useful sequence for the evaluation of cardiac masses because it not only provides both early localization of the mass, it also helps to detect the targets for subsequent sequences.

These images are acquired either with a free-breathing single-shot fast spin-echo (FSE) method (pulse sequence parameters include: slice thickness, 8–10 mm, no gap; usually 16– 20 slices; field of view, 320–400 mm; repetition time m sec/echo time m sec, 2,000/20; flip angle, 90°; matrix, 256 × 256) or with a breath hold FSE method (parameters include: slice thickness, 4–6 mm, no gap; usually 16–20 slices; field of view, 320–400 mm; repetition time m sec/echo time m sec, 1,000/40; flip angle, 90°; matrix, 512 × 512).

If the patients can tolerate it, a breath-hold FSE sequence with reduced slice thickness is ideal because of higher spatial-resolution. Higher spatial resolution improves assessment of anatomical consideration as it provides detailed information about relationships with surrounding structures as well as local invasion of the mass (16).

#### 2.1.3. Cine imaging

Cine images are used to further confirm the location (Figure 1) and morphology of a particular cardiac mass. They also allow assessment of the mobility and any functional impairment such as contractile dysfunction or obstruction to flow due the lesion (17).

Cine imaging with steady state free precession (SSFP) sequences (pulse sequence parameters include slice thickness, 8 mm, no gap; 3.2/1.6; flip angle, 60°; sensitivity encoding factor 31.7–2.0; 30–50 phases per cardiac cycle; field of view, 320–400 mm; matrix, 192 × 192) is the current ideal process for a cardiac mass imaging protocol. Tissue contrast with SSFP sequences is dependent on the T2/T1 ratio of the tissue. Therefore, tumors that have the same T2/T1 ratio as blood are not very well visualized with these sequences.

Cine imaging in the standard long axes and short axis stacks should be acquired to provide complete coverage of ventricles. In a specific region where the mass is suspected, slice thickness should also be reduced (4–6-mm, no gap) to obtain higher spatial resolution. This technique is of particular significance in the evaluation of small or highly mobile lesions. In addition to the standard cine images, nonstandard imaging planes should also be obtained through the mass in at least two orthogonal planes to further visualize the extent of the mass in various directions.

#### 2.1.4. Myocardial tissue tagging

Myocardial tissue tagging is an optional sequence that can be acquired if there is suspicion of myocardial infiltration or pericardial invasion of the cardiac mass. It may be helpful to detect more subtle areas of contractile impairment. It is often used for the evaluation of malignant cardiac masses that involve or invade the pericardium.

## 3. Tissue characterization

Cardiac masses are initially suspected on TTE and CMR is further performed to differentiate tumors from pseudo-tumors and to further characterize the cardiac masses based on their appearance on T1/T2-weighted images, detection of perfusion and demonstration of gadolinium contrast agent uptake on LGE images. Different cardiac tissues have different T1 and T2 relaxation times, principally owing to different internal biochemical environments surrounding the protons; therefore, in CMR, the signal intensity from a particular tissue depends on its T1 and T2 relaxation times and its proton density. Based on this principle, CMR can describe the differences in the signal intensity to differentiate between different tissue types and weight images based on their T1 or T2 relaxation time. Generally, tumor cells are larger, edematous, have associated inflammatory reactions and increased interstitial fluid. Higher free water content of the neoplastic cells and other changes in tissue composition lead to prolonged T1/T2 relaxation times and thus an inherent contrast between tumors and normal tissue exists (18). Overall, these biochemical changes lead to an environment where each cardiac mass produces different signal intensity on T1-weighted and T2- weighted images that helps to discriminate between different cardiac masses.

### 3.1. T1-Weighted imaging

These are double inversion recovery (IR), T1-weighted FSE image sequences (pulse sequence parameters include: slice thickness, 6–8 mm, no gap; repetition time m sec/echo time m sec, 1,000/40; flip angle, 90°; matrix, 512 × 512), which are obtained to completely cover the cardiac mass. These images are usually performed with and without a fat saturation spectral pre-saturation inversion recovery (SPIR) pulse. The SPIR technique is an extremely valuable method of characterizing fat containing lesions such as lipomas. T1 Weighted images may also be repeated approximately 15 s after the contrast agent injection to further characterize the cardiac mass. Malignant and highly vascular lesions (e.g., hemangioma) demonstrate significant post-contrast enhancement. Additional T1 Weighted FSE saturation sequence across the superior and inferior vena cava (IVC) may be considered to reduce the signal from slowly flowing blood. The downside of such a scan is an increase in the total scan time.

### 3.2. T2-Weighted imaging

These are double or triple IR, T2-weighted, FSE image sequences, which are obtained before the contrast material injection and should be acquired in the same imaging planes as set by T1-weighted images (sequence parameters include: slice thickness, 6–8 mm, no gap; repetition time m sec/echo time m sec, 2,000/100; flip angle, 90°; inversion time, 160 m sec; matrix, 192 × 192). T2-weighted FSE images are very helpful for the detection of regions of edema or Liquefactive necrosis in the mass, which have high signal intensity. While regions of coagulative necrosis have low signal intensity on these images (19). In evaluation of intracardiac thrombus, T2-weighted images help to identify the scattered areas of hemorrhage within the thrombus that usually have high signal intensity.

### 3.3. First-pass perfusion (FPP) imaging

These are optional rest perfusion sequences, which may be considered to determine the vascularity of a cardiac mass. Typically, 0.05–0.1 mmol/kg of gadolinium-based contrast agent is used to acquire these images. Vascular tumors (e.g., angiosarcoma) usually demonstrate significant enhancement during the arterial phase, whereas nonvascular masses (e.g., thrombus) do not enhance. These are also useful to describe the regions of heterogeneous enhancement owing to variations in the regional vascularity of a cardiac mass.

### 3.4. Early gadolinium enhancement (EGE) imaging

These are double IR, T1-weighted, FSE image sequences with longer inversion times (450,600 ms) and are obtained for identification of cardiac thrombus (pulse sequence parameters include: slice thickness, 8 mm, no gap; 4.5/1.8; flip angle, 15; matrix, 240 × 240) and ideally should be performed in those imaging planes where the tumor is best visualized. Typically, EGE imaging is performed within the first 2 min of 0.1–0.2 mmol/kg of gadolinium-based contrast agent infusion. Therefore, a “top-up” bolus may be needed if first-pass perfusion imaging is to be performed.

Characteristically, cardiac mass representing a thrombus does not pick up contrast material, and therefore almost always appears black on EGE images, whereas the blood pool and myocardium may show intermediate signal intensity on these images. In rare instances, neovascularization of the thrombus causes a reticular enhancement pattern on EGE images.

### 3.5. Late gadolinium enhancement (LGE) imaging

These are double IR, T1-weighted, FSE images with optimal inversion time (TI) for optimal nulling of the myocardium, which is set by T1-scout, “Look locker” or modified look locker inversion recovery (MOLLI) sequences (pulse sequence parameters include: slice thickness, 8 mm, no gap; 4.5/1.8; flip angle, 15; matrix, 240 × 240). The TI must be identified on an individual basis as it may vary from patient to patient. Optimal selection of the TI is critical to create the ideal image for diagnosis. If the myocardium is not properly nulled because the inversion time is too short, then the myocardium appears streaky or patchy. If the inversion time is too long, then the myocardium appears gray and it becomes difficult to differentiate between mass and myocardium.

Normal TI is generally around 300 m sec that largely depends on the patient's cardiac output and time after contrast agent injection. TI requires constant adjustment as the scan progresses to allow contrast washout of the normal myocardium; 10 m sec increments of change in the TI after every 1–3 slices is usually appropriate. Phase sensitive inversion recovery (PSIR) methods are also available which do not require a T1 scout.

Typically, these images are obtained 7–10 min after the injection of contrast material that usually consists of a 20 ml bolus of 0.1 mmol/kg of gadolinium contrast injection followed by a 20 ml normal saline bolus. Acquisition can also be repeated with a longer delay time to better characterize the abnormalities within the mass and to enhance the contrast between the blood pool and the mass.

The general scheme for complete myocardial coverage includes a complete short-axis stack and one to three sections of both long axes. After completion of the general scheme, imaging planes that allow for specific characterization of the mass are performed.

Historically, this technique has been used to detect viable myocardium after myocardial infarction (MI). The basic principle is that the contrast agent washes out of the normal myocardium over time but persists in any condition that expands the interstitial space. These conditions include but are not limited to acute inflammation (myocarditis), fibrosis, and MI (20).

Another pathophysiological model proposes that the cellular breakdown that occurs in acute MI or direct tumor invasion allows gadolinium to become intracellular, and therefore its concentration increases in the necrotic myocardium. A combination of the above factors most likely accounts for the demonstration of late gadolinium enhancement of cardiac masses on CMR. Cardiac tumors frequently have variable components of necrosis and fibrosis; specific enhancement patterns of the various tumor types are described in relative sections.

### 3.6. T1 mapping

T1 mapping is a novel CMR technique that has shown great utility in various cardiac pathologies, especially in myocardial fibrosis. There has not been a great deal of work done to understand the utility of this technique for tissue characterization of cardiac masses. Despite low numbers (*N* = 30), post-contrast T1 mapping has shown promising results in differentiating thrombus from tumors in a recent study by Lopez et al. (21). They demonstrated an increment in post- contrast T1 time with thrombus compared with tumors (477 ± 139 vs. 383 ± 84). On the other hand, post contrast T1 time was not significantly different between benign and malignant tumors (370 ± 63 vs. 386 ± 91). Relative absence of fibrotic content and extracellular matrix in thrombotic tissue compared with neoplastic tissue can solely explain these findings. However, it may be challenging in chronic organized thrombus, which may have some fibrotic material and it seems likely that there will be less difference in T1 time of thrombus and neoplasm in this scenario. Therefore, larger studies are required to further validate the impact of this novel technique in tissue characterization of cardiac masses.

## 4. Benign tumors

### 4.1. Myxoma

Myxomas are the most common type and account for approximately 30%–50% of primary benign cardiac tumors (Table 2). They usually occur in the fourth to seventh decade of life and almost 70% of them occur in women (22). Morphologically, they appear as well defined, smooth surfaced, lobulated, round masses with a narrow pedicle (23). Myxomas are typically highly mobile due a pedunculated attachment, but they can also be relatively fixed with broad-based features and a frond-like appearance. They are solitary in nature, typically have a predilection for the fossa ovalis of the interatrial septum and range from 1 to 15 cm in size. Majority of the cases of myxomas develop in the atria. Out of those cases, almost 75% (Figure 1) occur in the left atrium (LA) and 25%–30% occur in the right atrium (RA). Rarely, they can also develop in both atria and ventricles (5%–10%). Sometimes, multiple myxomas can also be seen particularly in association with Carney's triad (an autosomal dominant syndrome consisting of multiple cardiac myxomas, various pigmented skin lesions such as blue nevi, and extracardiac tumors.

Myxomas can be asymptomatic if they are small, but most of them present with at least one of the features of the so-called “myxoma triad” consisting of symptoms due to mass effect, thromboembolic phenomenon or constitutional symptoms (24). Cardiac myxomas are highly mobile structures and they frequently protrude into the respective atrioventricular valve during diastole and therefore they can potentially cause obstruction. LA myxomas can cause obstruction of the mitral valve and can mimic mitral stenosis and present with dyspnea and orthopnea. On the other hand, RA myxomas can obstruct the tricuspid valve and present with right heart failure symptoms.

Systemic or pulmonary embolization occurs in 30%–40% of the patients and cerebral circulation is most often affected (25). Patients can also present with constitutional symptoms such as fever, arthralgia, or rash due to release of pro-inflammatory mediators (i.e., interleukin 6). Most of the cases of symptomatic myxomas are treated with surgical resection to prevent further major complications. The overall recurrence rate is approximately 13% and mostly occurs in familial cases. Annual follow-up with TTE is recommended for a period of minimum 4 years because most of the cases of recurrence occur at the site of the original tumor within 4 years (26).

#### 4.1.1. Cardiac MRI features

**Figure 3:**
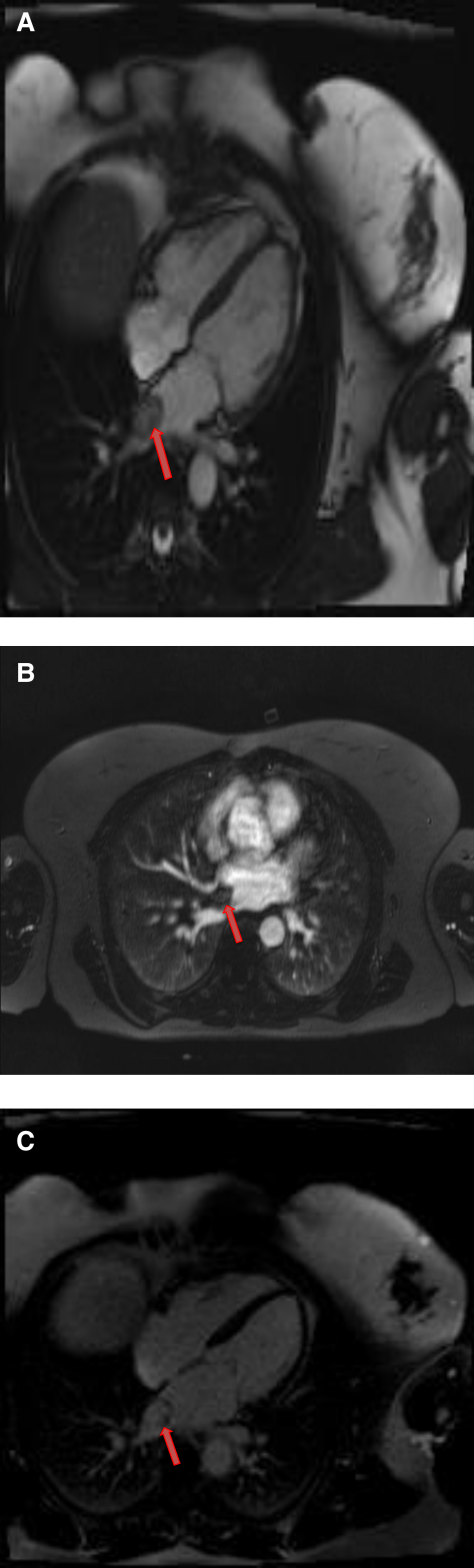
A 51-year-old female with left atrial myxoma. (**A**) Absence of perfusion of the lesion on first pass perfusion images (red arrow). (**B**) Following gadolinium administration, there is brisk, heterogeneous enhancement of this lesion on late gadolinium enhanced images (red arrow).

**Figure 4:**
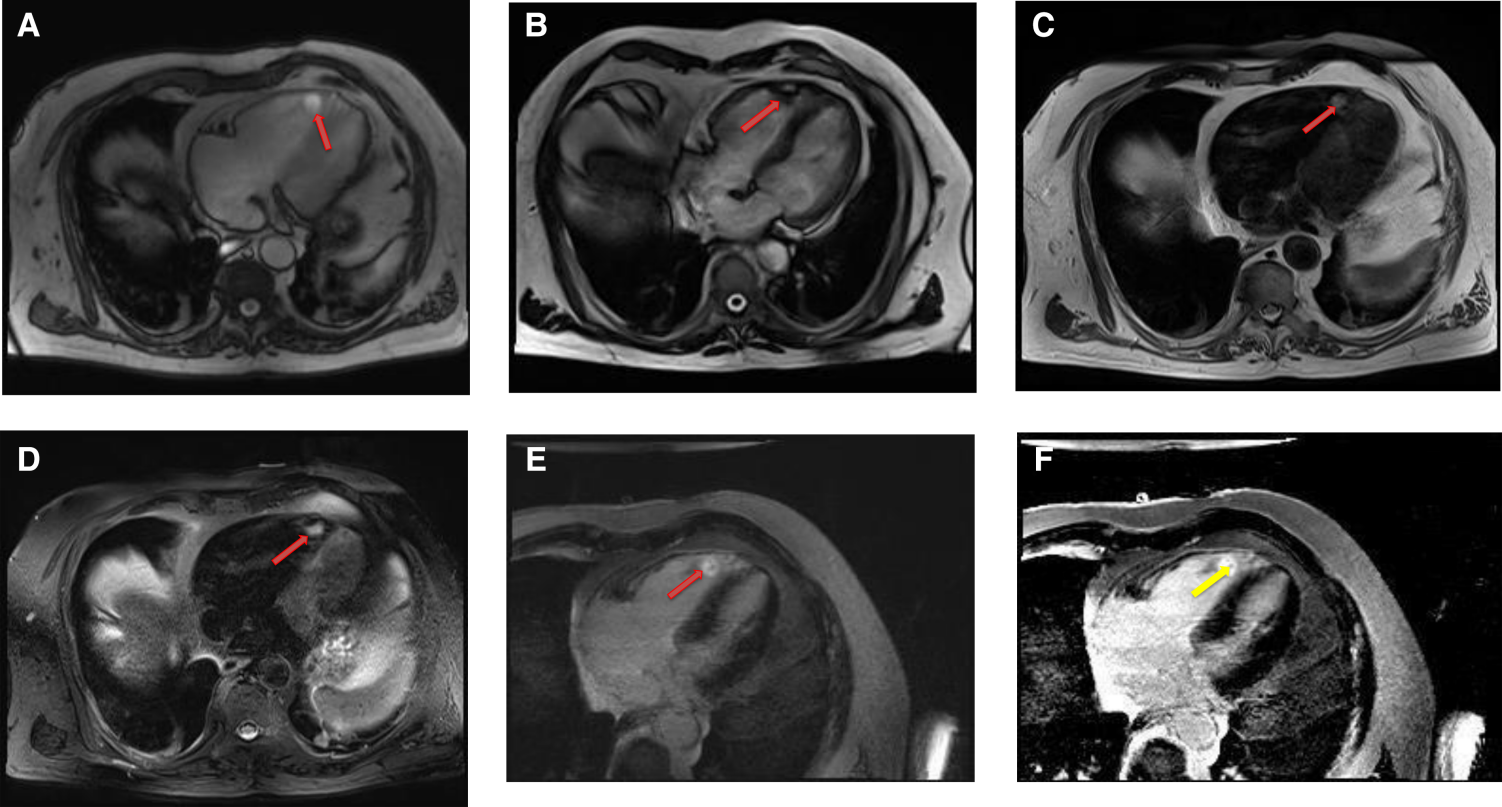
A 69-year-old female with right ventricular apical myxoma. (**A**) Localizer CMR images showing a small hyperintense mass in the distal RV (red arrow). (**B**) The mass showing high signal intensity on axial T2weighted, dark blood, double inversion recovery fast spin echo images (red arrow). (**C**) The mass shows absence of perfusion on first pass perfusion images (red arrow). (**D**) Heterogeneous enhancement of this lesion is noted on late gadolinium enhanced images. (**E**) There are small central areas of non-enhancement representing necrotic or hemorrhagic material (yellow arrow). RV, right ventricle.

Cardiac myxomas typically have a heterogeneous appearance on CMR because of varying components of myxoid, cystic, hemorrhagic, fibrotic, and calcified material (27). As they are highly mobile tumors, cine imaging is often very helpful in the work-up of myxomas. Tumor mobility, including mitral valve prolapse causing obstruction, may be seen on the cine images. On SSFP cine sequences myxomas are usually hypointense relative to the blood pool and hyperintense relative to the myocardium (28). They are isointense/hypointense to the myocardium on T1- weighted images and hyperintense on T2-weighted images likely due to high extracellular water contents. There may be associated regions of necrosis and hemorrhage that appear hypointense on both T1- and T2-weighted images (29). Most of the myxomas demonstrate at least some mild patchy enhancement on the first pass perfusion images. Myxomas usually show moderate to high, heterogeneous enhancement on LGE images (Table 1 and Figures 3, 4). Occasionally myxomas may also exhibit a homogeneous enhancement pattern.

Intracardiac thrombus, which is the major differential diagnosis for myxomas can be easily differentiated based on complete absence of both first pass perfusion and late gadolinium enhancement. However, it is not uncommon that many myxomas may have a layer of surface thrombus that typically show low signal on LGE images (30).

### 4.2. Lipoma

Lipomas are the second most common type (15%–20%) of primary cardiac tumors (Table 2) and mainly occur at a young age. They are slow growing, well defined, homogeneous, encapsulated tumors containing the neoplastic adipose tissue (31). Lipomas arising from the subendocardial layer are generally smaller, whereas large sized lipomas arise from the subepicardial or mid-myocardial layers (32) (Figure 1). Most of the patients are diagnosed incidentally and remain asymptomatic, but they can also present with atrial arrhythmias (33).

Rarely, large tumors may cause obstructive symptoms due to mass effects resulting in sudden cardiac death (22). On TTE cardiac lipomas can have highly variable appearances, which make them difficult to differentiate from myxomas or other tumors.

**Figure 5:**
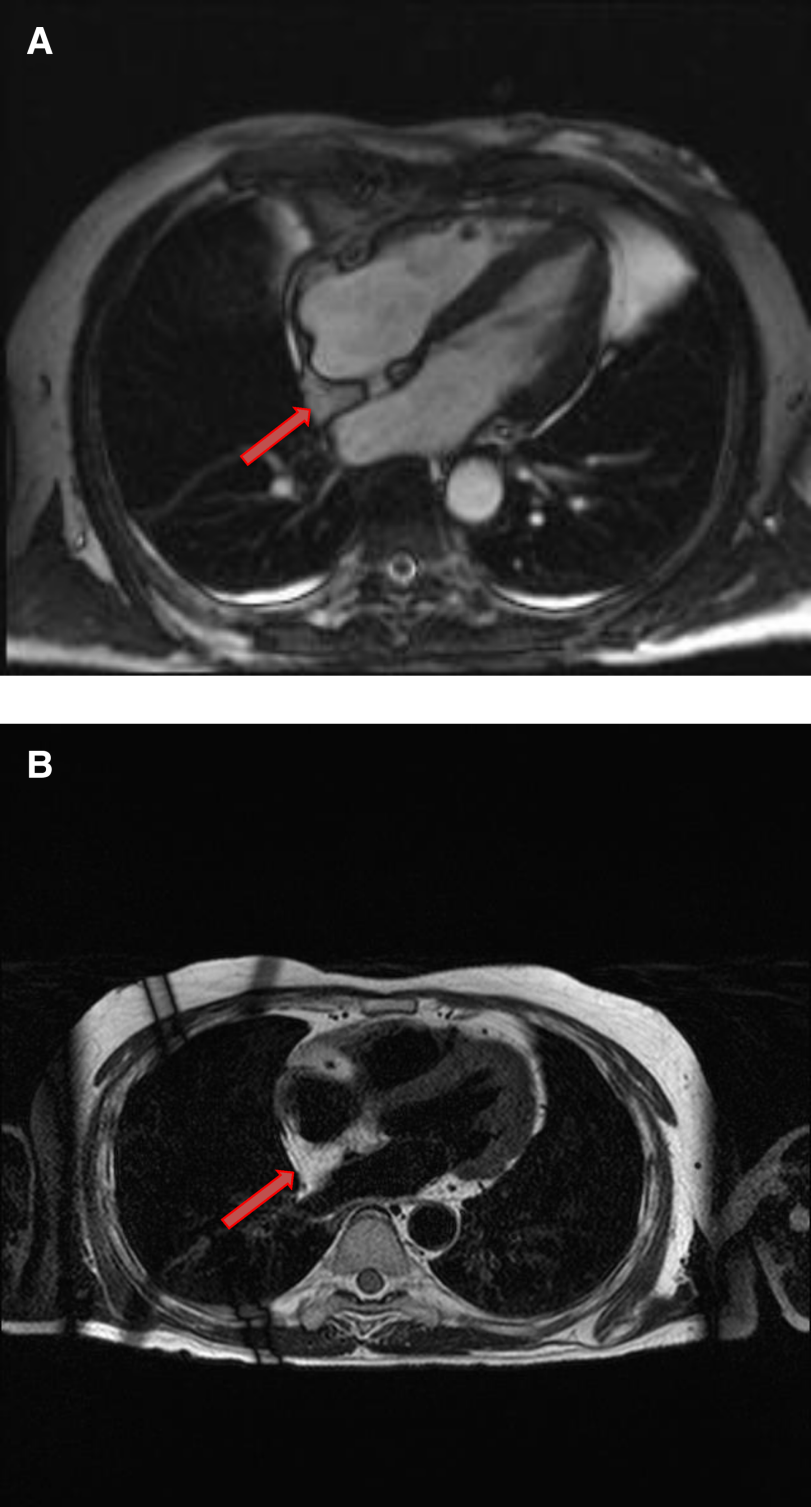
A 53-year-old male with lipomatous hypertrophy of the intra-atrial septum. (**A**) Cine CMR images showing diffuse enlargement and fatty replacement of the intra-atrial septum, consistent with lipomatous hypertrophy of the intra-atrial septum (red arrow). (**B**) Characteristic “dumbbell shaped” appearance of the mass on axial dark blood, double inversion recovery fast spin echo images (red arrow).

**Figure 6:**
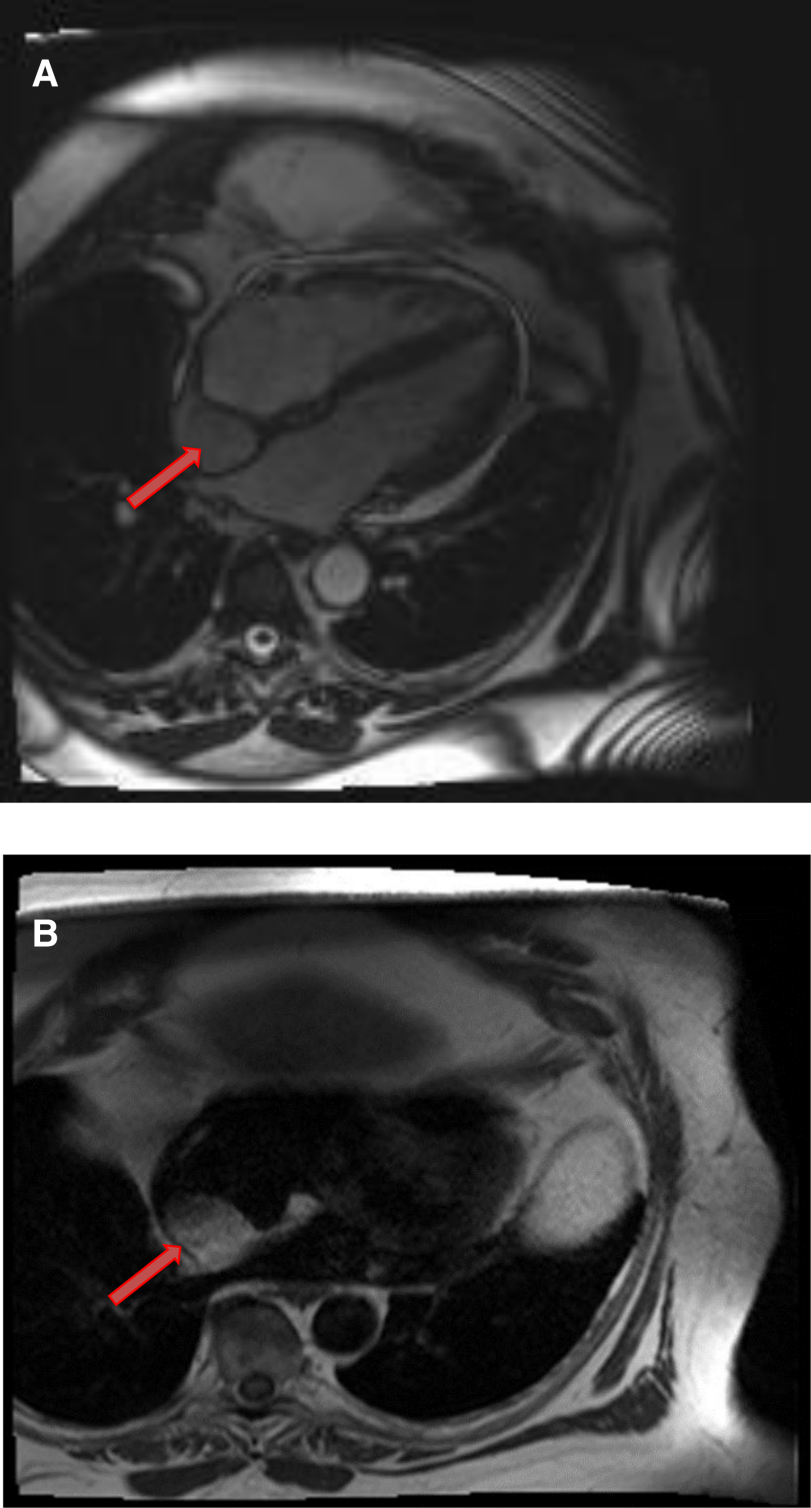
A 61-year-old male with lipomatous hypertrophy of the intra-atrial septum. (**A**) Cine CMR images showing diffuse enlargement and fatty replacement of the intra-atrial septum, consistent with lipomatous hypertrophy of the intra-atrial septum (red arrow). (**B**) Characteristic “dumbbell shaped” appearance of the mass on axial dark blood, double inversion recovery fast spin echo images (red arrow).

Lipomatous hypertrophy of the interatrial septum is a benign, non-encapsulated, nonneoplastic condition characterized by adipose cell hyperplasia in that region and is often found in older, overweight individuals (34). Lipomatous hypertrophy has the same signal characteristics as lipoma due to their fat content (35). However, lipomatous hypertrophy can be differentiated from lipoma by its morphologic features including larger than 2 cm size, typical involvement of the limbus of fossa ovalis that give rise to a characteristic “bilobed dumbbell appearance” (36) (Figures 5, 6). Most of the patients remain asymptomatic, but there are reported cases of atrial arrhythmias.

#### 4.2.1. Cardiac MRI features

**Figure 7:**
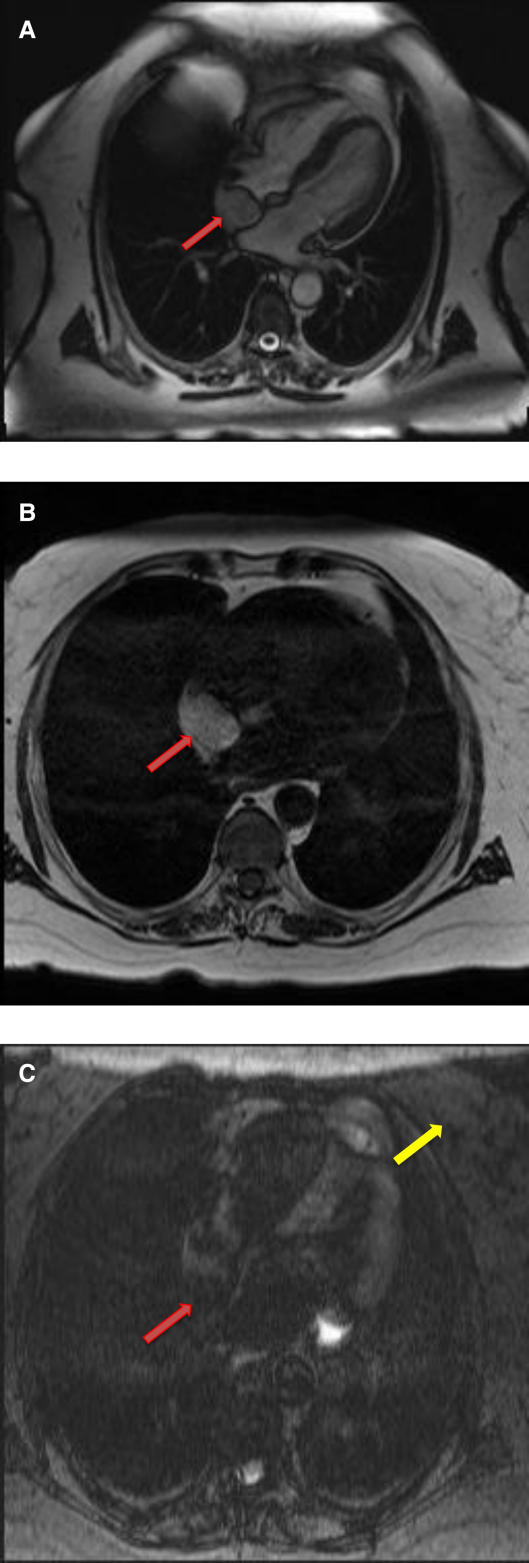
A 68-year-old female with intra-atrial septal lipoma. (**A**) (On line video 3), CMR cine images showing a sharply marginated mass arising from the intraatrial septum (red arrow). (**B**) This mass has same signal intensity (high) as surrounding fat on axial T1-weighted, dark blood, double inversion recovery fast spin echo images (red arrow). (**C**) Fat suppression sequence representing suppression of surrounding fat (white arrow) as well as the mass in the intra-atrial septum (red arrow).

**Figure 8:**
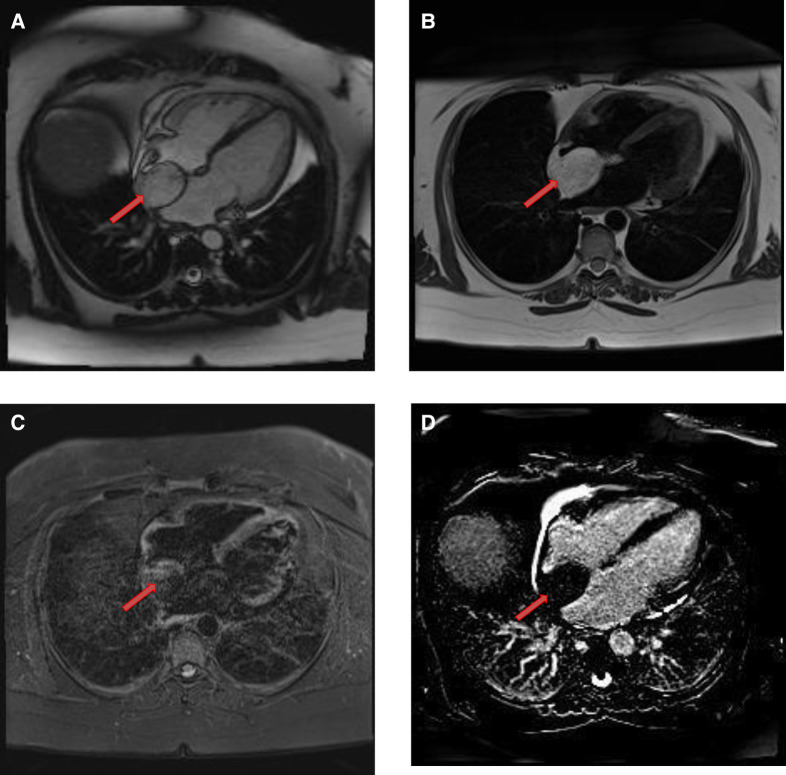
A 50-year-old female with intra-atrial septal lipoma. (**A**) This mass has signal intensity (high) as surrounding fat on axial T2-weighted, dark blood, double inversion recovery fast spin echo images (red arrow). (**B**) This mass is suppressed on axial triple inversion recovery images (red arrow). (**C**) Complete absence of contrast agent uptake on late gadolinium enhanced images (red arrow).

CMR provides a definitive diagnosis of both lipoma and lipomatous hypertrophy. Lipomas typically have a homogeneous appearance on CMR. Characteristically, they have the same signal intensity as surrounding chest wall fat on both T1- and T2-weighted images. Additionally, two extremely useful sequences are pre and post contrast fat saturated T1 Weighted sequences. Characteristically, signal dropout is observed on these sequences confirming the diagnosis of a fat-containing lesion. Due to their avascular nature, they do not show contrast enhancement on LGE images (Table 1 and Figures 7, 8).

### 4.3. Fibroelastoma

Papillary fibroelastomas (PFE's) are the third most common (10%–15%) benign cardiac tumor (Table 2) followed by atrial myxomas and lipomas (37). More than 80% of these tumors are located on the valvular endocardium and most commonly involve either aortic or mitral valve (Figure 1). Most non-valvular PFEs are also confined to the left ventricle. Estimated incidence of right atrial PFE is around 1.8%–2.3% (38). Reviewed the reported cases of non-valvular right atrial PFE, and most of these cases were diagnosed incidentally during routine cardiac workup due to unrelated symptoms (39).

TTE is the initial test of choice and has sensitivity of 88.9% and specificity of 87.8%. TEE is more sensitive than TTE if tumor size is less than 20 mm. Other diagnostic modalities are CT and gadolinium enhanced CMR. Histologically, PFEs have a characteristically gross appearance described as “sea anemone-like” branching tumors with multiple papillary fronds arising from a central stalk once placed under water. Under microscopy each frond shows an avascular core of elastin and collagen fibers lined by a flat endocardium.

All symptomatic patients should be evaluated for surgical resection. Surgery is recommended even in asymptomatic patients if the tumor size is more than 10 mm because fibroelastomas have tendency to embolize over a period of time (40). Vegetations and thrombi are the main differential diagnoses. Vegetation's are usually associated with the clinical context of infective endocarditis and cause destruction of valvular leaflets, whereas valvular incompetence is rarely present in fibroelastomas. Thrombus can be easily discriminated against on the basis of complete absence of LGE on CMRI.

#### 4.3.1. Cardiac MRI features

**Figure 9:**
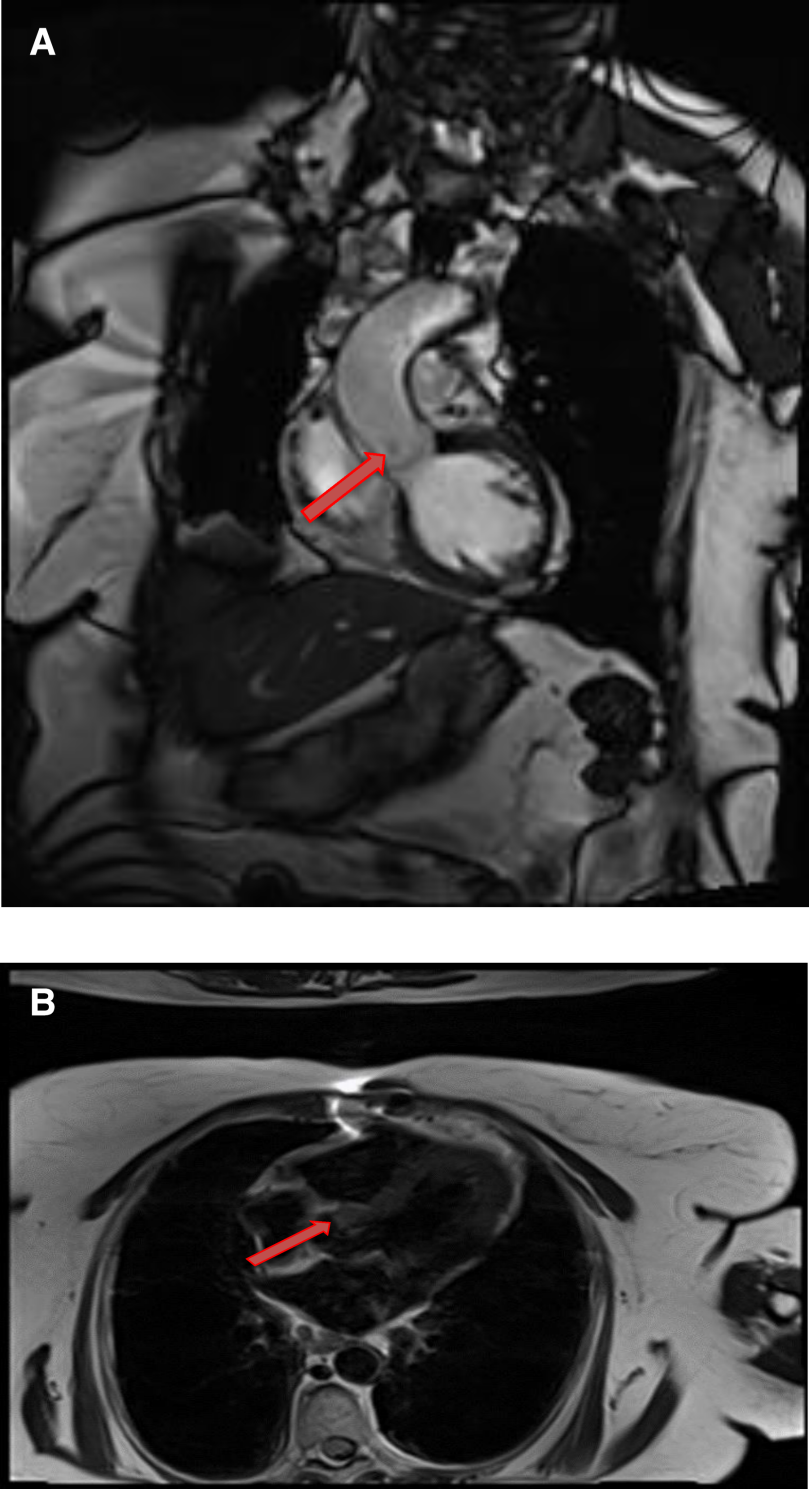
A 71-year-old female with aortic valve fibroelastoma. (**A**) This mass showing hyper-intense signal on T2weighted, dark blood, double inversion recovery fast spin echo images (red arrow).

PFE's typically appear as small, round, highly mobile, homogeneous valvular masses (usually attached to the downstream side with a small pedicle) on CMR. On SSFP cine sequences, PEFs are usually hypointense with a turbulent flow signal surrounding them. Compared to the myocardium, they are isointense on T1-weighted images and hyperintense on T2-weighted images. PEFs are differentiated from lipomas by fat saturation sequences, the former do not show any signal dropout. LGE is usually absent but uniform enhancement has also been described, presumably due to accumulation of gadolinium within fibroelastic tissue (41) (Table 1 and Figures 9).

### 4.4. Rhabdomyoma

Majority of the benign primary cardiac tumors in infancy and childhood are rhabdomyomas that generally present in the first year of life (42). Most commonly they develop from the ventricular myocardium in the form of single or multiple masses (Figure 1). Unlike fibromas, majority of the cases present with multiple rhabdomyomas and are strongly associated with tuberous sclerosis. Microscopically, they are hamartomas and have a pathognomonic “spider like” appearance representing degenerating rhabdomyocytes (43). Most of the cases remain asymptomatic and undergo spontaneous regression by the age of 4 years. Surgery is only recommended in symptomatic patients with heart failure or arrhythmias (44, 45). Diagnosis is usually made by TTE; cardiac MRI is reserved for atypical cases or prior to surgical intervention (46).

#### 4.4.1. Cardiac MRI features

**Figure 10:**
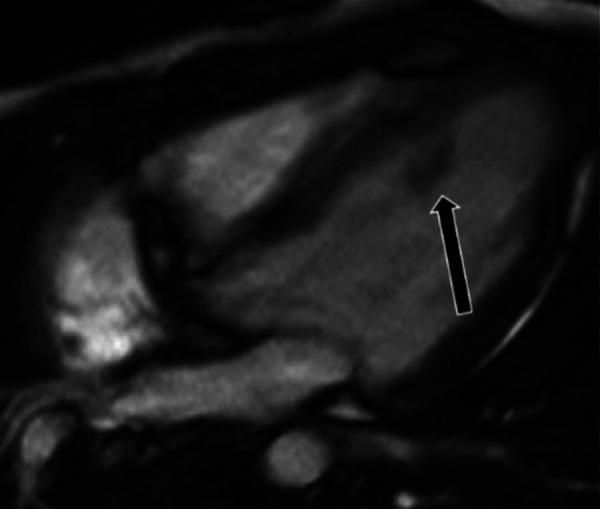
A 31-year-old male with left ventricular fibroma. (**A**) This mass demonstrates no perfusion on first-pass imaging (red arrow, short axis view). (**B–F**) Homogeneous intense enhancement of the mass is noted on late gadolinium enhanced images (red arrows, 3chamber and short axis views).

Rhabdomyomas are typically homogeneous in appearance on CMR and are hypointense to the myocardium on SSFP cine sequences. They appear isointense/hypointense on T1 Weighted images and slightly hyperintense on T2-weighted images (Figure 10). On LGE images, they usually show very minimal or no enhancement after the injection of contrast material (Table 1).

### 4.5. Fibroma

Fibromas are the second most common congenital tumor and account for almost 5% of benign cardiac tumors (Table 2). Majority of them are detected in the pediatric population or occasionally in young adults. Most commonly they occur in the interventricular septum (Figure 1) and involve the left ventricle more than the right ventricle (47).

Rarely, cardiac fibromas can be associated with polyposis syndromes, and those fibromas are more commonly located in the atria (48). Although histologically benign, clinically they can present with palpitations, arrhythmias, syncope, chest pain, heart failure, outflow tract obstruction, or even sudden cardiac death.

Morphologically, they are well-defined, solitary lesions within the myocardium involving the inter ventricular septum. Microscopically, cardiac fibromas are aggregates of collagen and neoplastic fibroblastic cells with significant central calcification that differentiates them from rhabdomyomas. Even asymptomatic fibromas are treated with surgical resection because of the risk of sudden cardiac arrest (49).

#### 4.5.1. Cardiac MRI features

Fibromas are of variable signal intensity on SSFP cine images. As compared to the myocardium, they are usually isointense on T1-weighted images and are characteristically hypointense on T2-weighted images (50). Although, they are generally homogeneous in appearance, a patchy, central hypo intensity may be seen due to central calcification, which gives them a heterogeneous appearance.

They are avascular in nature and do not show contrast enhancement during perfusion imaging. Characteristically, they show intense hyper-enhancement on LGE images. The potential explanation of this intense LGE pattern is that microscopically they are composed of collagen and fibroblasts; therefore, they have a very large extracellular space content. Gadolinium diffuses into interstitial spaces and results in a delayed and persistent concentration of gadolinium contrast agent (Table 1 and Figure 10).

### 4.6. Hemangioma

Hemangiomas are highly vascular cardiac tumors and represent 3%–5% (Table 2) of primary benign cardiac tumors (51). They are usually solitary and can arise from any cardiac chamber, however they are more common in the ventricles (Figure 1 and in Figure 13). In histology, they can be cavernous, capillary, or arteriovenous in nature. Most of the patients are asymptomatic, but rarely they can present with exertional dyspnea (52).

#### 4.6.1. Cardiac MRI features

Hemangiomas are typically heterogeneous in appearance on CMR, presumably due to calcification and fibrous septa. They are isointense or hyperintense on T1- weighted images because of slow blood flow and they are hyperintense on T2- weighted images. Owing to high vascular contents, they show significant enhancement during and after gadolinium contrast administration, which may be heterogeneous. An exception is cavernous hemangioma that may not show significant LGE owing to very slow blood flow (53) (Table 1).

### 4.7. Other rare benign tumors

Teratomas are usually located intrapericardial and commonly affect the infants as shown in Figure 14. A pericardial effusion generally accompanies them. Diagnosis is made by CMR, and surgical resection is usually recommended (54).

**Figure 11:**
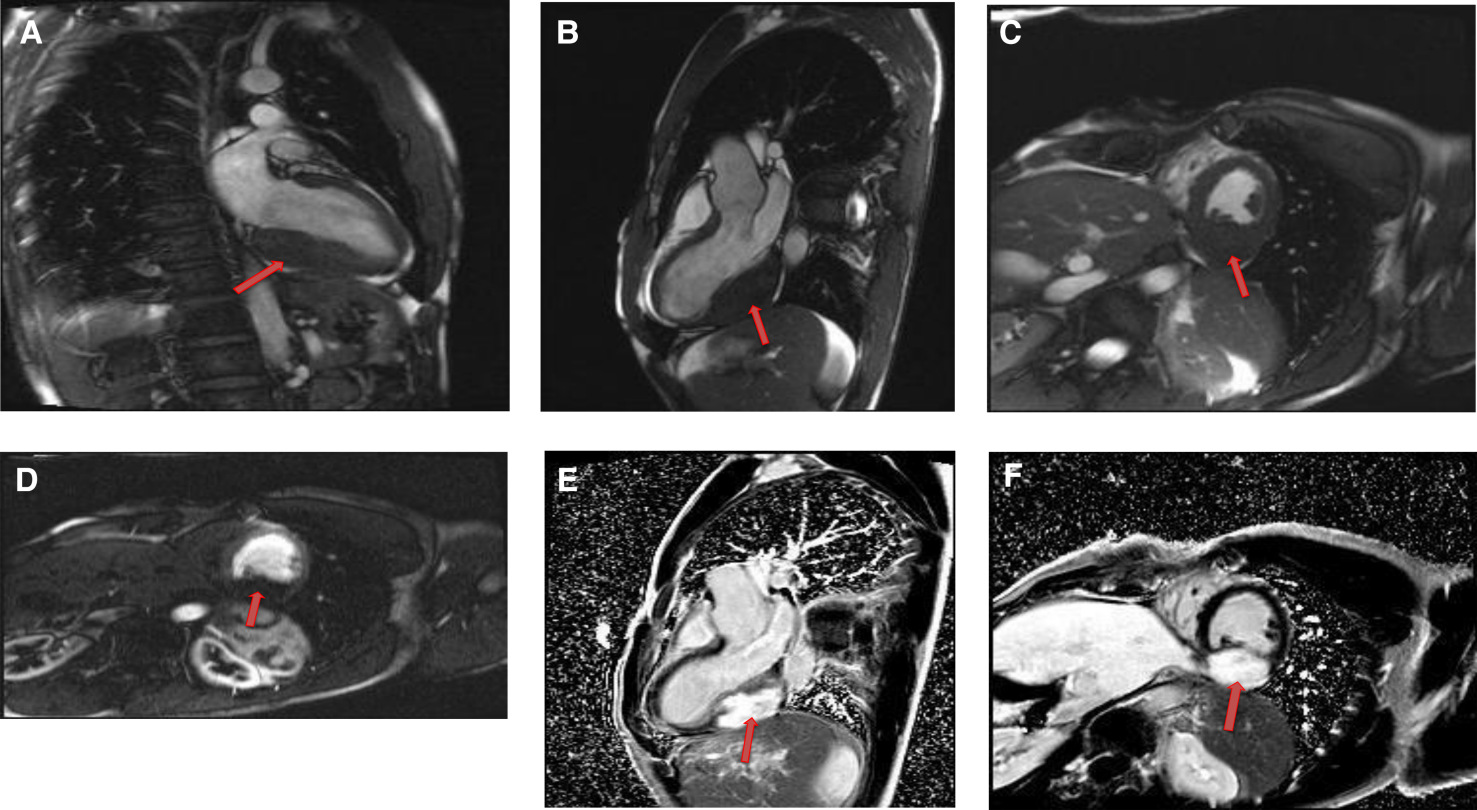
A 54-year-old male with right ventricular pheochromocytoma. (**A**) The mass is isointense on T1- weighted, dark blood, double inversion recovery fast spin echo images (red arrow). (**B**) The mass demonstrates peripheral hyper-vascularity with central areas of nonenhancement on first pass perfusion images (red arrow). (**C**) There is central enhancement of this mass on late gadolinium enhanced images (red arrow).

**Figure 12:**
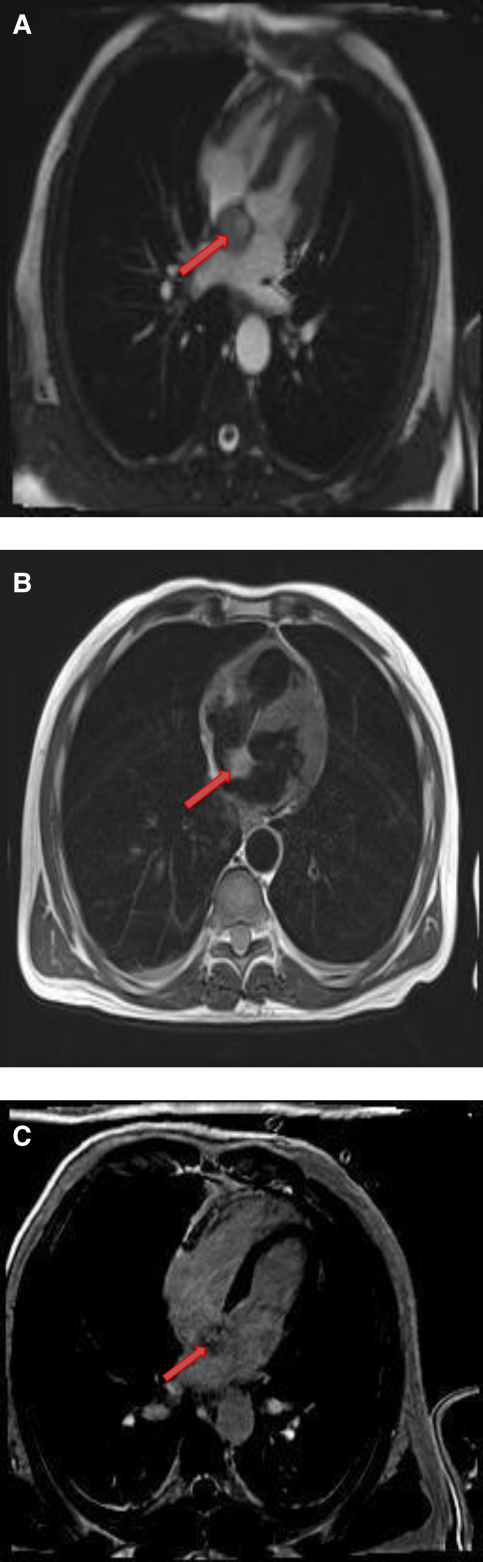
A 53-year-old male with inter-atrial septal pheochromocytoma. (**A**) The mass is bright on T2- weighted, dark blood, double inversion recovery fast spin echo images (red arrow). (**B**) This mass shows heterogeneous enhancement on late gadolinium enhanced images (red arrow).

Cardiac paragangliomas are rare hyper-vascular masses that arise from the cardiac neuroendocrine cells (55). Patients are usually young and present with various symptoms of excessive catecholamine secretion. Surgical resection is often recommended, although it is challenging presumably due to their vascular nature and intimate relationship with the coronary arteries. On CMR, they are isointense to the myocardium on T1-weighted images and significantly hyperintense on T2 -weighted images (56). Due to high vascularity, they typically show uniform, intense enhancement during and after gadolinium contrast administration (Figures 11,12). Occasionally, they may show heterogeneous enhancement due to associated areas of hemorrhage and necrosis.

**Figure 13:**
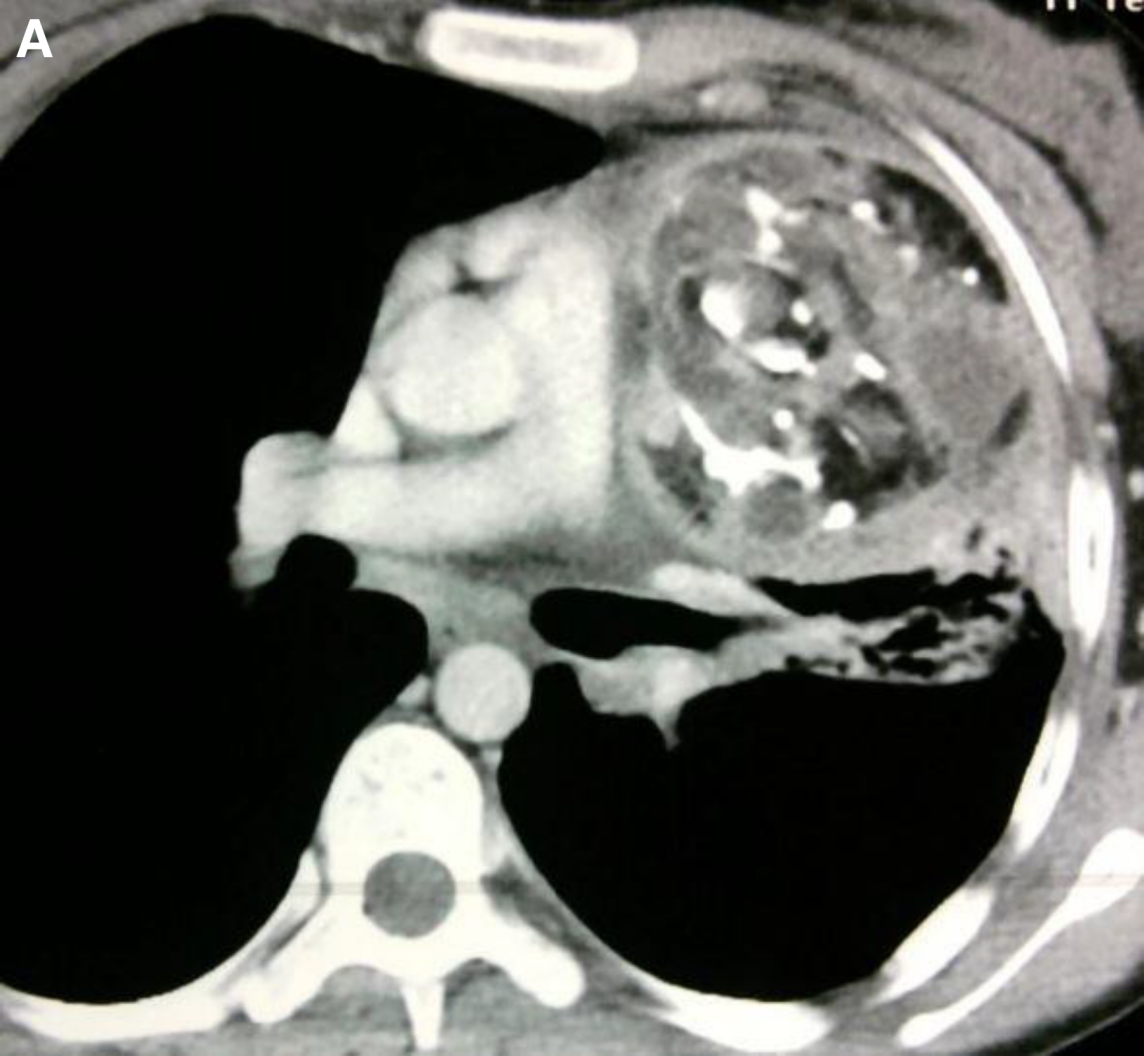
Cardiac Hemagioma in a 40 years old male. In Right ventricle there is round contrast enhancing lesion, involving the right ventricular cavity shown in Figure A is cardiac hemangioma.

**Figure 14:**
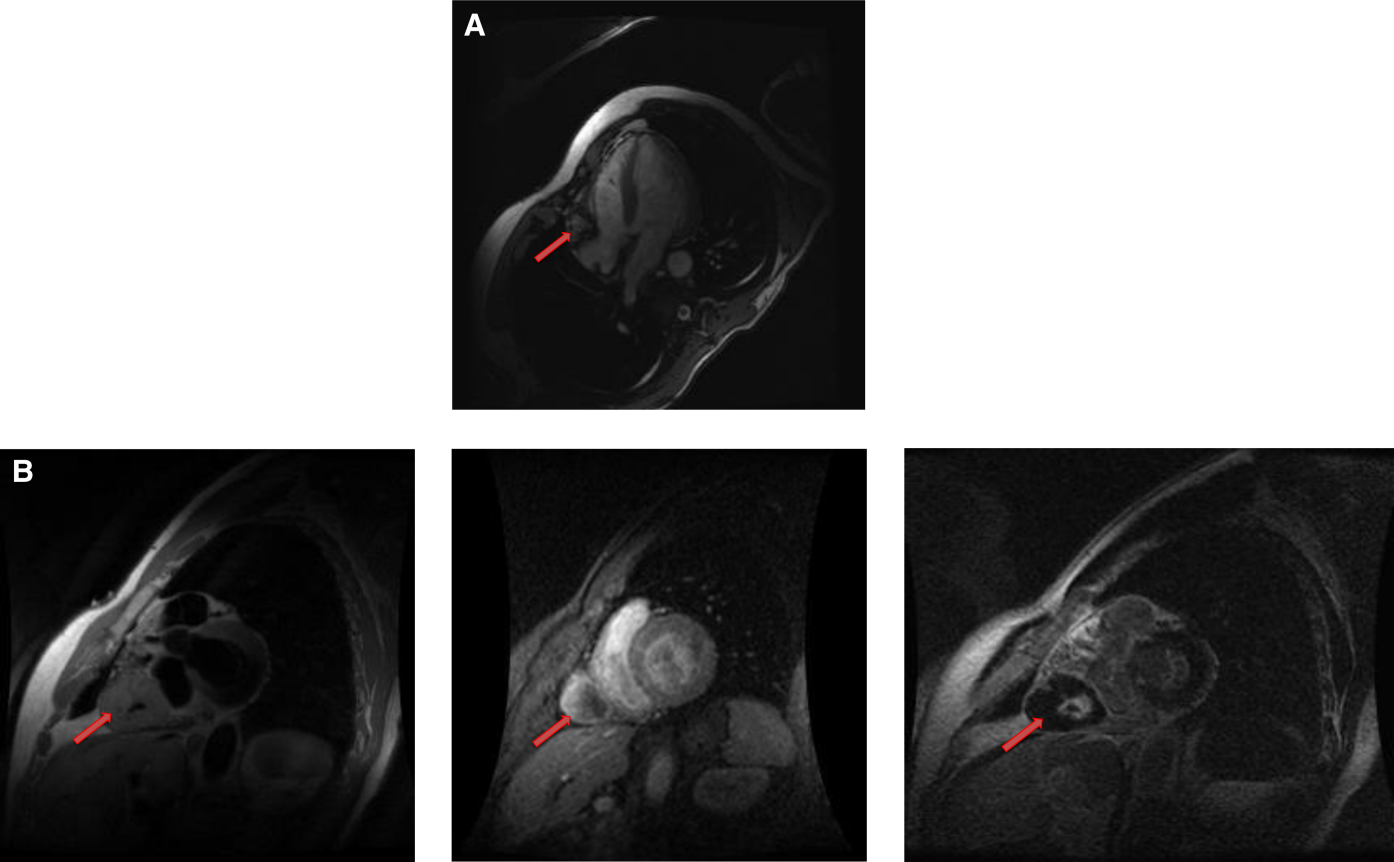
A mature teratoma in a 16 years old female patient. (**A**). Contrast enhancing CT chest showing a calcified, fatty lesion inside heart, calcifications are multifocal representing huge mature cardiac teratoma.

IV leiomyomatosis is a benign tumor of the smooth muscles. Rarely it has been reported to extend into the IVC and invade the right side of the heart (57).

## 5. Conclusions

Due to tremendous advancements in the field of cardiac imaging, CMR has been established as an extremely valuable tool in workup of suspected cardiac tumors. As compared with other imaging modalities, CMR provides unrestricted field of view, versatile imaging planes, excellent contrast resolution and superior tissue characterization without the risk of ionizing radiation. In the first part of this review article, we have described a detailed imaging protocol for evaluation of a suspected cardiac tumor that allows comprehensive assessment of the morphology, anatomy, tissue characterization as well as functional consequences of the mass.

In this part, we have also discussed benign cardiac tumors and the role of CMR in evaluation and further tissue characterization of these tumors. In summary (Table 4), most of the benign cardiac tumors are hypointense/isointense on T1-weighted images and hyperintense on T2-weighted images with the exception of fibromas that are hypointense on T2-weighted images. Benign tumors show homogeneous signal composition; only myxomas and hemangiomas demonstrate heterogeneous signal composition. Contrast agent uptake on LGE images among different benign tumors is variable. Highly vascular tumors like cardiac hemangiomas demonstrate enhancement on FPP images.

**Table 4:** CMR tissue characterization of benign tumors.

		Exceptions
T1-weighted imaging	Isointense/Hypointense	
T2-weighted imaging	Hyperintense	Fibroma
LGE imaging	Variable contrast uptake	
Signal composition	Homogeneous	Myxoma hemangioma

CMR, cardiac magnetic resonance; LGE, late gadolinium enhancement.

## References

[1] Sparrow PJ Kurian JB Jones TR MR Imaging of cardiac tumors. Radiographic. (2005) 25:1255–76. 10.1148/rg.255045721 16160110

[2] Sütsch G Jenni R von Segesser L Schneider J. Heart tumors: incidence, distribution, diagnosis exemplified by 20,305 echocardiographies. Schweiz Med Wochenschr. (1991) 121:621–29. PMID: 2047823 2047823

[3] Bruce CJ. Cardiac tumors: diagnosis and management. Heart. (2011) 97:151–60. 10.1136/hrt.2009.186320 21163893

[4] Elbardissi AW Dearani JA Daly RC Survival after resection of primary cardiac tumors: a 48-year experience. Circulation. (2008) 118:S7–15. 10.1161/CIRCULATIONAHA.107.783126 18824772

[5] Roberts WC. Primary and secondary neoplasms of the heart. Am J Cardiol. (1997) 80:671–82. 10.1016/S0002-9149(97)00587-0 9295010

[6] Butany J Nair V Naseemuddin A Nair GM Catton C Yau T. Cardiac tumors: diagnosis and management. Lancet Oncol. (2005) 6:219–28. 10.1016/S1470-2045(05)70093-0 15811617

[7] Gupta S Plein S Greenwood JP. The coumadin ridge: an important example of a left atrial pseudotumour demonstrated by cardiovascular magnetic resonance imaging. J Radiol Case Rep. (2009) 3:1–5. 10.3941/jrcr.v3i9.210 22470681 PMC3303338

[8] Araoz PA Mulvagh SL Tazelaar HD Julsrud PR Breen JF. CT And MR imaging of benign primary cardiac neoplasms with echocardiographic correlation. Radio Graphics. (2000) 20:1303–19. 10.1148/radiographics.20.5.g00se121303 10992020

[9] Mandegar MH Rayatzadeh H Roshanali F. Left atrial myxoma: the role of multislice computed tomography. J Thorac Cardiovasc Surg. (2007) 134:795. 10.1016/j.jtcvs.2007.05.034 17723837

[10] O’Donnell DH Abbara S Chaithiraphan V Cardiac tumors: optimal cardiac MR sequences and spectrum of imaging appearances. Am J Roentgenol. (2009) 193:377–87. 10.2214/AJR.08.1895 19620434

[11] Delbeke D Coleman RE Guiberteau MJ Procedure guideline for tumor imaging with 18F-FDG PET/CT 1.0. J Nucl Med. (2006) 47:885–95. PMID: 16644760 16644760

[12] Bluemke DA Achenbach S Budoff M Non- invasive coronary artery imaging: magnetic resonance angiography and multi-detector computed tomography angiography. A scientific statement from the American heart association committee on cardiovascular imaging and intervention of the council on cardiovascular radiology and intervention, and the councils on clinical cardiology and cardiovascular disease in the young. Circulation. (2008) 118:586–06. 10.1161/CIRCULATIONAHA.108.189695 18586979

[13] Naehle CP Strach K Thomas D Magnetic resonance imaging at 1.5-T in patients with implantable cardioverter-defibrillators. J Am Coll Cardiol. (2009) 54:549–55. 10.1016/j.jacc.2009.04.050 19643318

[14] Sutton R Kanal E Wilkoff BL Safety of magnetic resonance imaging of patients with a new Medtronic EnRhythm MRI SureScan pacing system: clinical study design. Trials. (2008) 9:68. 10.1186/1745-6215-9-68 19055703 PMC2629460

[15] Spuentrup E Mahnken AH Kühl HP Fast interactive real time magnetic resonance imaging of cardiac masses using spiral gradient echo and radial steady state free precession sequences. Invest Radiol. (2003) 38:288–92. 10.1097/01.RLI.0000064784.68316.34 12750618

[16] Stehling MK Holzknecht NG Laub G Böhm D von Smekal A Reiser M. Single- shot T1- and T2-weighted magnetic resonance imaging of the heart with black blood: preliminary experience. MAGMA. (1996) 4:231–40. 10.1007/BF01772011 9220412

[17] Kramer CM Barkhausen J Flamm SD Kim RJ Nagel E. Society for cardiovascular magnetic resonance board of trustees task force on standardized protocols. Standardized cardiovascular magnetic resonance imaging (CMR) protocols, society for cardiovascular magnetic resonance: board of trustees task force on standardized protocols. J Cardiovasc Magn Reson. (2008) 10:35. 10.1186/1532-429X-10-35 18605997 PMC2467420

[18] Mitchell DG Burk DL Jr Vinitski S Rifkin MD. The biophysical basis of tissue contrast in extracranial MR imaging. Am J Roentgenol. (1987) 149:831–7. 10.2214/ajr.149.4.831 3307357

[19] Simonetti OP Finn JP White RD Laub G Henry DA. “Black blood” T2-weighted in- version-recovery MR imaging of the heart. Radiology. (1996) 199:49–57. 10.1148/radiology.199.1.8633172 8633172

[20] Kim RJ Wu E Rafael A The use of contrast enhanced magnetic resonance imaging to identify reversible myocardial dysfunction. N Engl J Med. (2000) 343:1445–53. 10.1056/NEJM200011163432003 11078769

[21] Pazos-López P Pozo E Siqueira ME Value of CMR for the differential diagnosis of cardiac masses. JACC Cardiovasc Imaging. (2014) 7:896–05. 10.1016/j.jcmg.2014.05.009 25129516

[22] Restrepo CS Largoza A Lemos DF CT And MR imaging findings of benign cardiac tumors. Curr Probl Diagn Radiol. (2005) 34:12–21. 10.1067/j.cpradiol.2004.10.002 15644859

[23] Grebenc ML Rosado de Christenson ML Burke AP Primary cardiac and pericardial neoplasms: radiologic–pathologic correlation. Radiographics. (2000) 20:107303. 10.1148/radiographics.20.4.g00jl081073 10903697

[24] Grebenc ML Rosado-de-Christenson ML Green CE From the archives of the AFIP: cardiac myxoma: imaging features in 83 patients. RadioGraphics. (2002) 22:673–89. 10.1148/radiographics.22.3.g02ma02673 12006696

[25] El Bardissi AW Dearani JA Daly RC Survival after resection of primary cardiac tumors: a 48-year experience. Circulation. (2008) 118:S7–S15. 10.1161/CIRCULATIONAHA.107.783126 18824772

[26] Donsbeck AV Ranchere D Coindre JM Primary cardiac sarcomas: an immunehistochemical and grading study with long-term follow-up of 24 cases. Histopathology. (1999) 34:295–04. 10.1046/j.1365-2559.1999.00636.x 10231396

[27] Luna A Ribes R Caro P Evaluation of cardiac tumors with magnetic resonance imaging. Eur Radiol. (2005) 15:1446–55. 10.1007/s00330-004-2603-y 15627179

[28] Schvartzman PR White RD. Imaging of cardiac and paracardiac masses. J Thorac Imaging. (2000) 15:265–73. 10.1097/00005382-200010000-00006 11039614

[29] Matsuoka H Hamada M Honda T Morphologic and histologic characterization of cardiac myxomas by magnetic resonance imaging. Angiology. (1996) 47:693–8. 10.1177/000331979604700709 8686964

[30] Basso C Valente M Poletti A Casarotto D Thiene G. Surgical pathology of primary cardiac and pericardial tumors. Eur J Cardiothorac Surg. (1997) 12:730–7. 10.1016/S1010-7940(97)00246-7 9458144

[31] Hananouchi GI. Goff WB 2nd. Cardiac lipoma: six-year follow-up with MRI characteristics, and a review of the literature. Magn Reson Imaging. (1990) 8:825–8. 10.1016/0730-725X(90)90021-S 2266812

[32] Braunwald E Zipes DP Libby P. Heart disease. Philadelphia, PA: Saunders (2001). 1807–19.

[33] Friedberg MK Chang IL Silverman NH Near sudden death from cardiac lipoma in an adolescent. Circulation. (2006) 113:e778–9. 10.1161/CIRCULATIONAHA.105.589630 16735681

[34] Heyer CM Kagel T Lemburg SP Bauer TT Nicolas V. Lipomatous hypertrophy of the interatrial septum: a prospective study of incidence, imaging findings, and clinical symptoms. Chest. (2003) 124:2068–73. 10.1378/chest.124.6.2068 14665481

[35] Suarez-Mier MP Fernandez-Simón L Gawallo C. Pathologic changes of the cardiac conduction tissue in sudden cardiac death. Am J Forensic Med Pathol. (1995) 16:193–02. 10.1097/00000433-199509000-00002 7495258

[36] Pugliatti P Patane S De Gregorio C Recupero A Carerj S Coglitore S. Lipomatous hypertrophy of the interatrial septum. Int J Cardiol. (2008) 130:294–5. 10.1016/j.ijcard.2007.07.012 17714811

[37] Gowda RM Khan IA Nair CK Mehta NJ Vasavada BC Sacchi TJ. Cardiac papillary fibroelastoma: a comprehensive analysis of 725 cases. Am Heart J. (2003) 146:404–10. 10.1016/S0002-8703(03)00249-7 12947356

[38] Sun JP Asher CR Yang XS Clinical and echocardiographic characteristics of papillary fibroelastomas: a retrospective and prospective study in 162 patients. Circulation. (2001) 103:2687–93. 10.1161/01.CIR.103.22.2687 11390338

[39] Atalay MK Taner AT. Gradual enhancement of a large left atrial papillary fibroelastoma on cardiac magnetic resonance: the waiting game. Tex Heart Inst J. (2010) 37:612–3. PMID: 20978584 20978584 PMC2953232

[40] Nwiloh J Hernandez E Mercado A. Right atrial papillary fibroelastoma. J Card Surg. (2011) 26:39–41. 10.1111/j.1540-8191.2010.01042.x 20459455

[41] Kelle S Chiribiri A Meyer R Papillary fibroelastoma of the tricuspid valve seen on magnetic resonance imaging. Circulation. (2008) 117:e190–1. 10.1161/CIRCULATIONAHA.107.729731 18347215

[42] Beghetti M Gow RM Haney I Mawson J Williams WG Freedom RM. Pediatric primary benign cardiac tumors: a 15-year review. Am Heart J. (1997) 134:1107–14. 10.1016/S0002-8703(97)70032-2 9424072

[43] Burke A Virmani R. Tumors of the heart and great vessels. In: Tavora F, editor. Atlas of tumor pa- thology. 3rd series, fasc 16. Washington, DC: Armed Forces Institute of Pathology (1996). p. 171–9.

[44] Stiller B Hetzer R Meyer R Primary cardiac tumors: when is surgery necessary? Eur J Cardiothorac Surg. (2001) 20:1002–6. 10.1016/S1010-7940(01)00951-4 11675188

[45] Bielefeld KJ Moller JH. Cardiac tumors in infants and children: study of 120 operated patients. Pediatr Cardiol. (2013) 34:125–8. 10.1007/s00246-012-0399-0 22735896

[46] Cho JM Danielson GK Puga FJ Surgical resection of ventricular cardiac fibromas: early and late results. Ann Thorac Surg. (2003) 76:1929–34. 10.1016/S0003-4975(03)01196-2 14667615

[47] Yang HS Arabia FA Chaliki HP De Pe- tris G Khandheria BK Chandrasekaran K. Images in cardiovascular medicine. Left atrial fibroma in Gardner syndrome: real time 3dimensional transesophageal echo imaging. Circulation. (2008) 118:e692–6. 10.1007/s00059-005-2667-8 19001028

[48] Alkadhi H Leschka S Hurlimann D Jenni R Genoni M Wildermuth S. Fibroelastoma of the aortic valve: evaluation with echocardiography and 64-slice CT. Herz. (2005) 30:438. 10.1007/s00059-005-2667-8 16132248

[49] Hoffmann U Globits S Schima W Usefulness of magnetic resonance imaging of cardiac and paracardiac masses. Am J Cardiol. (2003) 92:890–5. 10.1016/S0002-9149(03)00911-1 14516903

[50] McAllister HA Jr. Primary tumors and cysts of the heart and pericardium. Curr Probl Cardiol. (1979) 4:1–51. 10.1016/0146-2806(79)90008-2 230012

[51] Thilak R Sivanesan A Munuswamy H A giant right atrial hemangioma- case report. Cureus. (2022) 14((4):e24622. 10.7759/cureus.24622 35664397 PMC9150831

[52] Oshima H Hara M Kono T Shibamoto Y Mishima A Akita S. Cardiac hemangioma of the left atrial appendage: CT and MR findings. J Thorac Imaging. (2003) 18:204–6. 10.1097/00005382-200307000-00012 12867820

[53] Kiaffas MG Powell AJ Geva T. Magnetic resonance imaging evaluation of cardiac tumor characteristics in infants and children. Am J Cardiol. (2002) 89:1229–33. 10.1016/S0002-9149(02)02314-7 12008185

[54] Serraj M Lakranbi M Ghalimi J Mediastinal mature teratoma with complex rupture into the lung, bronchus and skin: a case report. World J Surg Onc. (2013) 11:125. 10.1186/1477-7819-11-125 PMC3674925 23725382

[55] Sahdev A Sohaib A Monson JP Grossman AB Chew SL Reznek RH. CT and MR imaging of unusual locations of extra-adrenal paragangliomas (pheochromocytomas). Eur Radiol. (2005) 15:85–92. 10.1007/s00330-004-2412-3 15290072

[56] Reynen K. Cardiac myxomas. N Engl J Med. (1995) 333:1610–7. 10.1056/NEJM199512143332407 7477198

[57] Altinok D Yildiz YT Tacal T Karapinar K Eryilmaz M. MRI Of intravascular leiomyomatosis extending to the heart. Eur Radiol. (2000) 10:871. 10.1007/s003300051023 10823652

